# A patterned single layer graphene resistance temperature sensor

**DOI:** 10.1038/s41598-017-08967-y

**Published:** 2017-08-18

**Authors:** Benyamin Davaji, Hak Dong Cho, Mohamadali Malakoutian, Jong-Kwon Lee, Gennady Panin, Tae Won Kang, Chung Hoon Lee

**Affiliations:** 1 Department of Electrical and Computer Engineering, Marquette University, Milwaukee, WI USA; 2 School of Electrical and Computer Engineering, Cornell University, Ithaca, NY USA; 3 Department of Physics, Quantum-Functional Semiconductor Research Center, Nano Information Technology Academy, Dongguk University, Seoul, Korea; 4 Department of Nanostructure Technology, National Nanofab Center, Daejeon, Korea; 5 Institute for Microelectronics Technology and High Purity Materials, RAS, 142432 Chernogolovka, Moscow district Russia

## Abstract

Micro-fabricated single-layer graphenes (SLGs) on a silicon dioxide (SiO_2_)/Si substrate, a silicon nitride (SiN) membrane, and a suspended architecture are presented for their use as temperature sensors. These graphene temperature sensors act as resistance temperature detectors, showing a quadratic dependence of resistance on the temperature in a range between 283 K and 303 K. The observed resistance change of the graphene temperature sensors are explained by the temperature dependent electron mobility relationship (~T^−4^) and electron-phonon scattering. By analyzing the transient response of the SLG temperature sensors on different substrates, it is found that the graphene sensor on the SiN membrane shows the highest sensitivity due to low thermal mass, while the sensor on SiO_2_/Si reveals the lowest one. Also, the graphene on the SiN membrane reveals not only the fastest response, but also better mechanical stability compared to the suspended graphene sensor. Therefore, the presented results show that the temperature sensors based on SLG with an extremely low thermal mass can be used in various applications requiring high sensitivity and fast operation.

## Introduction

Graphene is a monolayer of carbon in a honeycomb lattice. Since being isolated from graphite by the micromechanical cleavage method in 2004, the physical properties of graphene have been studied extensively^1^. The observed superior physical properties of graphene, such as high electron mobility^2, 3^, mechanical strength^4, 5^, optical properties^6, 7^, and thermal conductivity^8, 9^, have attracted much attention from the research community for a variety of applications^10–12^. Although various physical properties of single-layer graphene (SLG) have been measured and predicted^5, 13, 14^, its thermal properties, such as thermal conductivity, have been continuously investigated to date^15^. The higher thermal conductivity of graphene in comparison with metals and carbon nanotubes (CNT)^16^ has led to active research opportunities in thermal management and energy storage applications^8, 17^. At nanoscale, the performance of electronic devices suffers from elevated temperatures as a result of self-heating, so graphene’s outstanding thermal properties are considered to be suitable for both instrumentation and integrated microelectronic applications^18^. Recently developed techniques for fabricating complex graphene structures in micro/nano scale^19–21^ make graphene a great candidate for temperature sensor applications due to its excellent electrical properties, outstanding mechanical strength, and high thermal conductivity.

To use graphene as a temperature sensor, the relationship between electrical resistance and temperature needs to be characterized to determine viable applications. A linear relationship between temperature and electrical conductivity can lead to use as a resistive temperature detector (RTD) like metals, while a non-linear relationship can lead it to be used like a thermistor, similar to ceramics or semiconductors. Increasing demands for faster operation speed, improved temperature measurement resolution, and miniaturization capabilities have triggered a search for new materials in temperature sensing applications, such as bolometers^22^ and biomedical sensors^23, 24^. In resistance-based temperature sensors, the initial resistance (typically based on 0 °C) and the temperature coefficient of resistance (TCR) are essential parameters for temperature measurement. Commercially available RTDs have a resistance range of 100 Ω to 1000 Ω at 0 °C. For most metals used in RTD applications, the TCR is about 10^−3^ C^−1^. A higher TCR value means a higher sensitivity. Thus, lowering the TCR or increasing the thermal mass of a sensor helps to reduce the noise, but lowers the sensitivity.

Although SLG has good electrical and thermal properties, no significant temperature sensing application has been studied. Meanwhile, reduced Graphene Oxide (r-GO) and hybrid graphene-GO membranes have been used as temperature sensors^18^. However, complicated carrier scattering by large densities of defects and residual oxygen contamination in GO and r-GO sheets limits their resistance based applications^25–27^. In this paper, we present micro-patterned SLGs placed on silicon dioxide (SiO_2_)/Si, a silicon nitride (SiN) membrane, and a Si wafer with etched rectangular pits for their use as temperature sensors. After describing the details of the fabrication processes, the device characteristics are investigated by using the 4-wire measurement technique to observe the relationship between electrical resistance and temperature on the SLG temperature sensors on the various substrates. Also, the effects of carrier scattering on the mobility of graphene on different substrates^28^ are taken into account to explain the observed results.

## Results and Discussion

### Fabrication of the Graphene Temperature Sensor

We adapted the process of chemical vapor deposition (CVD) growth of graphene and the transfer method on a substrate from Gunes *et al*.^29^. Graphene layers grown on Cu foils by the CVD method were transferred to a Si substrate covered with a 250 nm thick thermally grown SiO_2_ layer (The detailed procedures were described in the experimental section). Then, the synthesized graphene layers were characterized by Raman spectroscopy^30, 31^. The measured Raman spectrum clearly shows the G (~1598 cm^−1^) and 2D (~2712 cm^−1^) bands with the rarely observed D band near 1350 cm^−1^, which verifies that the synthesized graphene is a high quality layer. Also, mono-layer graphene was identified by the peak intensity ratio of the 2D band to the G band greater than one, and the full width at half maximum of 2D peak less than 40 cm^−1^ ^32–34^.

**Figure 1:**
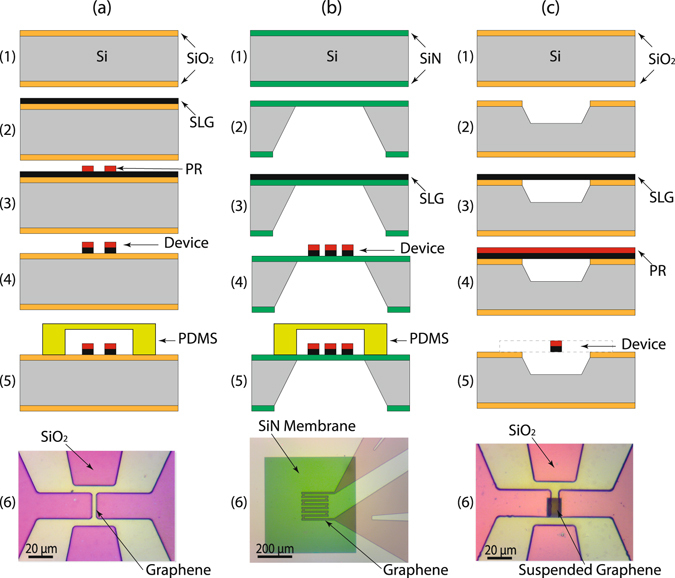
Micro-fabricated SLG temperature sensor devices on three different substrates. (**a**) SiO_2_/Si substrate. Cross-sectional view of the fabrication process flow including: thermal growth of SiO_2_ (1), SLG transferring (2), device patterning (3), plasma etching of graphene (4), and (5) PDMS gasket protection of the sensor during PR removal at electrical contacts area. (6) Close-up picture of the completed device with 4-wire electrodes configuration. (**b**) SiN substrate. Cross-sectional view of the fabrication process including: (1) LPCVD SiN deposition, (2) defining membrane using the KOH etching from the backside, (3) SLG transferring, (4) device patterning using O_2_ plasma and (5) PDMS gasket protection of the sensor during PR removal at electrical contacts area. (6) Close-up picture of the completed device with the 4-wire electrodes configuration. (**c**) Suspended graphene. Cross-sectional view of the fabrication process flow including (1) thermal growth of the SiO_2_, (2) patterning the suspension pits using BOE etching of SiO_2_ followed by KOH etching of Silicon, (3) SLG transferring, (4) spin coating of PR and (5) device patterning using O_2_ plasma. (6) Close-up picture of the completed device with the 4-wire electrodes configuration.

The graphene temperature sensors were fabricated on three different surfaces to investigate the scattering effects due to the graphene/surface boundaries on the resistance change as a function of temperature. The synthesized graphene layers were transferred to SiO_2_/Si, a SiN membrane, and a Si wafer with etched rectangular pits. After being transferred, the graphene was micro-patterned by a photo-lithography process as shown in Fig. 1(a), (b) and (c), respectively. In Fig. 1(a), a (100) Si wafer with a 250 nm thick thermally grown SiO_2_ film was used as a substrate. The CVD graphene layer was transferred to the substrate. Then, a thin layer of positive photoresist (PR) was spun, UV exposed, and developed. Then, the exposed graphene was removed by oxygen plasma at 300 W for 10 seconds and the resulting pattern is shown in Fig. 1(a.4). Here, a PDMS gasket was used to not only isolate the temperature sensing part from environmental factors, such as humidity and temperature fluctuation (Fig. 1(a.5)), but also to protect the temperature sensing area from the PR removal process, which was done with acetone to expose peripheral graphene for electrical contacts. The top view image of the completed device is shown in Fig. 1(a.6).

To investigate the surface boundary related scattering effects between graphene and the substrate, a graphene device was integrated on a low stress SiN membrane. The SiN membrane was fabricated using an anisotropic wet chemical etching (KOH) as explained previously^35–38^. Patterning the graphene device was similar to the graphene on the SiO_2_/Si. The graphene on the SiN membrane device was constructed with the fabrication process flow as shown in Fig. 1(b.1–5). The top view image of the completed device is shown in Fig. 1(b.6). In the SiN membrane case, the thermal mass of the device is reduced significantly; therefore, the temperature sensitivity is increased. Also, the thermal time constant of the device is shorter than that of the graphene device on the SiO_2_/Si substrate, resulting in a faster measurement. However, the surface roughness is much higher than that of SiO_2_/Si substrate.

Surface boundary scattering and defects play a key role in the electrical conductivity of graphene. To reduce the surface effects, a suspended SLG device was prepared. To fabricate the suspended graphene device, rectangular pits on a SiO_2_/Si substrate was prepared. The pits were fabricated by patterning rectangular boxes (10 × 20 *μ*m^2^ boxes) using photolithography on the SiO_2_/Si substrate. The patterned SiO_2_ was etched with a buffered oxide etchant followed by KOH silicon anisotropic etching. The SLG was transferred on the wafer with etched boxes, and the sensor patterns and electrical contacts were printed over the boxes. Then oxygen plasma at 300 W for 10 seconds was used to etch the exposed graphene as shown in Fig. 1(c.1–5). The oxygen plasma etch time was carefully selected to avoid etching the graphene layer underneath the patterned PR. As before, the temperature sensing part was protected using a PDMS gasket during the PR removal on the electrical contact pads using acetone to allow for electrical connections. The top view image of the suspended graphene sensor (PDMS gasket not shown) is shown in Fig. 1(c.6).

### Electrical Resistance Measurement

**Figure 2:**
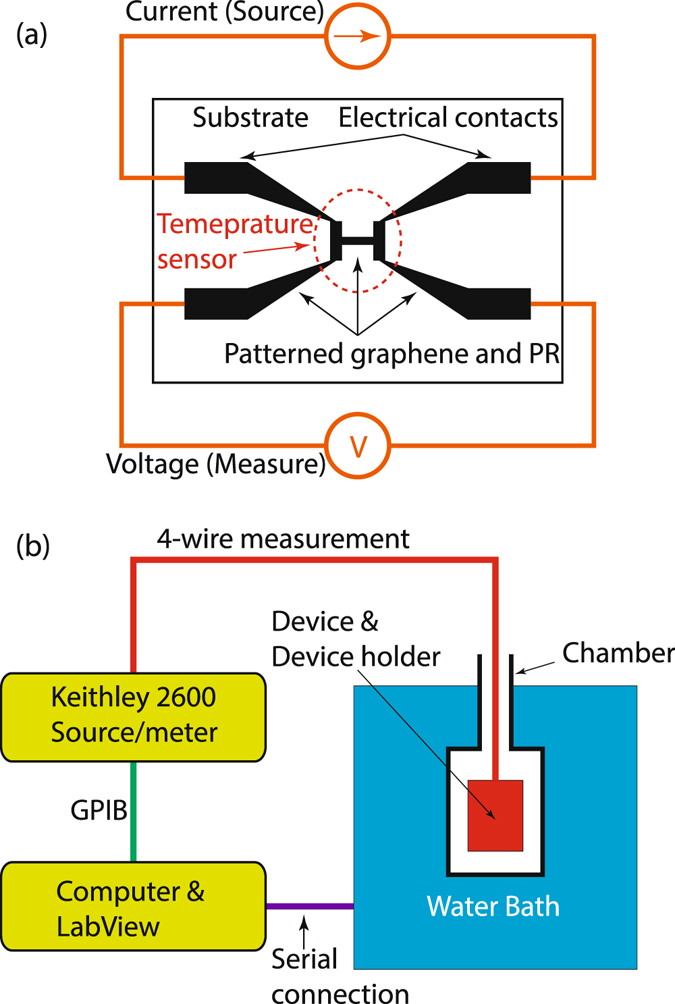
Measurement setup for characterizing the temperature response of micro-fabricated SLG temperature sensors. (**a**) Graphene device is displayed with a 4-wire resistance measurement setup. The device pattern design allows use of a 4-wire measurement to define the active temperature sensitive region of the device. (**b**) The temperature controlled test environment and measurement setup.

To measure the resistance of the micro-fabricated SLGs on the three different substrates, we used the 4-wire measurement technique^39^. A schematic of a graphene device as a temperature sensor is shown in Fig. 2(a). The temperature sensor part is indicated with a dotted circle in the middle of the device. Graphene is conductive enough to be used as the electrodes and electrical contacts for the resistance measurement. Using the graphene as a conductive trace and four contact pads to fan out from each device eliminates an extra metallization and patterning step which might increase the defects in the SLG. Four electrical graphene wires are connected to a current source and voltage meter for the 4-wire measurement. The 4-wire measurement is an accurate resistance measurement method because the resistances of the leads and electrodes have no effect on the measurement. The 4-wire measurement only measures the resistance or resistance change of the temperature sensor portion of the device. Since the 4-wire measurement uses a bias current source to create a voltage drop across the resistive graphene temperature sensor, the temperature sensor itself generates Joule heat, known as self-heating. To reduce the self-heating of the sensor, a bias current of 100 *μ*A was used throughout the experiments. The temperature increase due to self-heating at room temperature in this work was less than 0.01 °C. A temperature controlled chamber (water bath) was used to measure the temperature and resistance relationship of the graphene device as shown in Fig. 2(b). The temperature controlled water bath was chosen to increase the control over temperature and eliminate the effects of room temperature fluctuations and other external noise sources on the measurement. The water bath temperature was increased by 5 °C per 2 hours. The low rate of the temperature increase of the water bath is necessary due to the large thermal time constant of the chamber. The resistance of the patterned graphene (temperature sensor) was recorded continuously by 4-wire measurement. A custom made LabVIEW program controlled the temperature of the water bath and the sensor data was acquired from a Keithley 2600 source-meter.

### Resistance Change of Graphene Temperature Sensors

**Figure 3:**
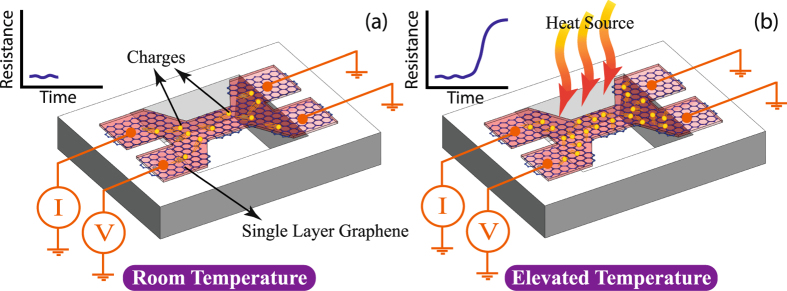
A schematic view of a graphene sensor. As the temperature increases from room temperature (**a**) to a high temperature (**b**), the number of electrons will be increased and their mobility will be decreased. The heat source in our measurement is the water bath as shown in Fig. 3(b).

The device resistance depends on the conduction electrons. By increasing the chamber temperature the number of electrons will be increased and their mobility will be decreased. A schematic view of the graphene device at low and high temperature is shown in Fig. 3.

**Figure 4:**
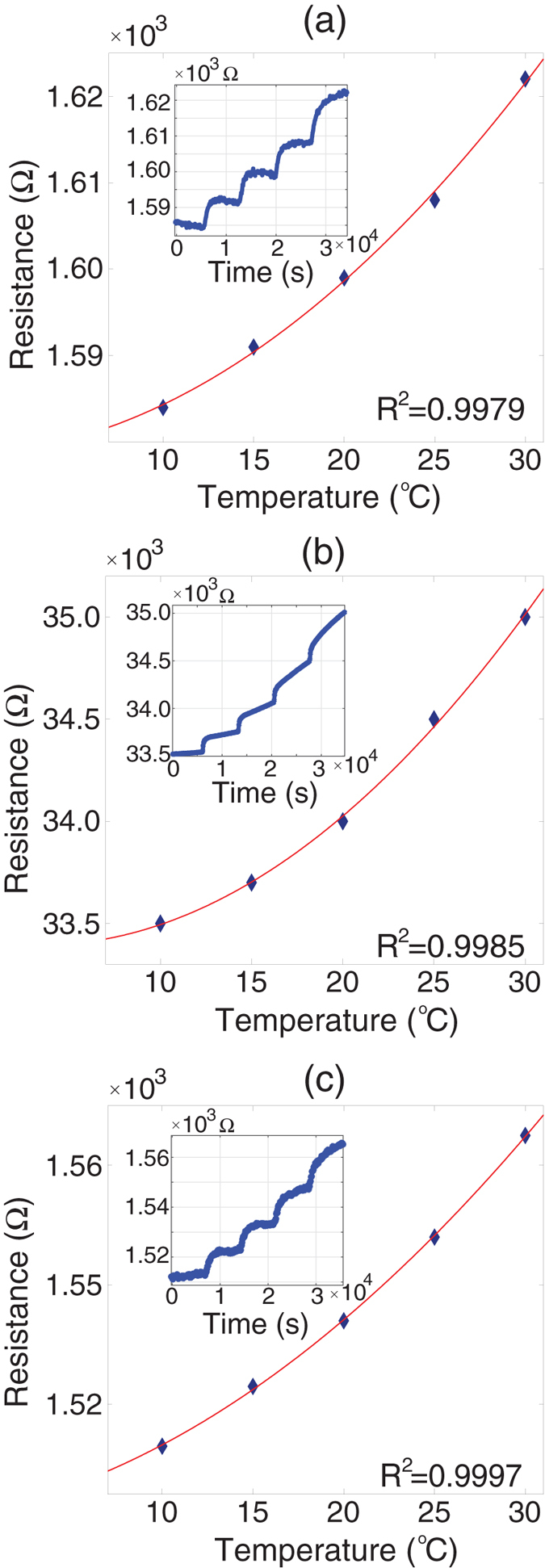
Resistance change as a function of time and temperature for the three different graphene sensors: (**a**) a graphene sensor on a SiO_2_/Si substrate, (**b**) a graphene sensor on a SiN membrane, and (**c**) the suspended graphene sensor (Y axis for all figures is resistance (Ω)).

The resistance change of the graphene devices in a temperature controlled chamber was measured. The water bath temperature was increased from 10 °C to 30 °C in 5 °C increments. The water bath temperature stayed at each temperature for 2 hours. The 2 hour dwell time at each temperature increment was used for the sensor inside the chamber to reach equilibrium with the water bath temperature. The measured results for the graphene devices on the SiO_2_/Si, the SiN membrane, and the suspended substrate are shown in Fig. 4(a), (b) and (c), respectively. The interior figures in Fig. 4 show the continuous change of the device resistance as a function of time. The main figures in Fig. 4 reveal the resistance values of the devices versus the water bath temperatures. The measured data was fitted with the coefficient of determination (R^2^) denoted by the red lines in Fig. 4, showing quadratic dependence of resistance on the temperature for all cases with R^2^ ≈ 1. While most metals show linear resistance change behavior as a result of temperature change, thermistors (most semiconductors) show non-linear (mostly exponential) resistance changes. In our result, the graphene resistor shows the resistance change is proportional to T^2^. The electrical conductivity of SLG^40^ is proportional to the temperature dependent carrier density and mobility of its electrons^11^: σ = 1/*ρ* = *qnμ*, where q is charge, n is the carrier density, and *μ* is the mobility of the mobile charge. The defects in graphene and the boundaries between the graphene and substrate contribute to carrier scattering, which are directly related to carrier mobility^3, 41, 42^. These defects can be introduced during the fabrication process, and different boundary conditions can be created by the varying geometry/size and substrate of the device. The significant contribution of interfacial scattering in graphene to electrical properties, like phonon-boundary scattering and graphene contact scattering, have been reported^2, 12, 43^. For the single layer graphene, it is reported that n is proportional to T^2^ ^11^. The mobility depends not only on temperature, but also on various scattering mechanisms, such as defects, phonons, surfaces, and edges^13, 25, 28, 44^. On the scale of our devices (micro-scale), it is a challenge to experimentally identify which scattering mechanism dominates and how it affects electron mobility as a function of temperature. According to previous numerical studies, the electron mobility of SLG considering various scattering mechanisms in a temperature range between 200 K and 350 K was reported to be proportional to T^−2^ ~ T^−4^ ^40^, and is limited by electron-phonon scattering in SLG^45^. The electron mobility in our device is deduced to be proportional to T^−4^ from the T^2^ dependent resistance in the range of 283 K to 303 K.

The resistance of graphene continues to increase at a fixed water bath temperature. It takes more than 2 hours for the resistance of the graphene to reach a steady state value in the water bath. This delay in reaching the steady state value may be due to the apparatus of the device holder. The device holder is made of bulky acrylic plates, which the entire device is mounted on. Since the thermal mass of the apparatus is high, it takes a long time (large thermal time constant) to reach a steady state value with the water bath temperature. The Si substrate is in thermal contact with the acrylic plate and quickly reaches the temperature of the acrylic plate. It takes more than 2 hours for the acrylic plate to reach the water bath temperature. Because graphene has a very high thermal conductivity^16^ and a very low thermal mass^46^, the temperature of graphene reaches the bulk silicon temperature quickly. The bulk silicon is in thermal equilibrium with the acrylic device holder and the air inside the chamber (Fig. 2(b)). Although the temperature of the graphene is slightly different from the water bath temperature, the temperature dependence of graphene resistance (~T^2^) is valid, because the temperature equilibrium characteristics between the apparatus and graphene are the same for each temperature of the water bath. The temperature dependent resistance of graphene is measured from 10 °C to 30 °C because the apparatus starts to physically distort above 35 °C. It was an unexpected experimental fact. The physical distortion of the apparatus applies a mechanical stress on the graphene, causing resistance fluctuations. This response to the mechanical stress shows that graphene is a piezoresistor. The piezoresistive properties of the graphene causes a proportional resistance change due to the applied deformation and strain^47–49^.

In addition, in Fig. 4 the sensitivity can be expressed as dR/dT, resistance change (response) per temperature change (external). The sensitivity is obtained from the slope of the resistance measurement over temperature data. The data is not linear, but we can take an average of resistance change after normalizing the resistance. The normalized resistance sensitivities for the devices on the SiO_2_/Si, the SiN membrane, and the suspended architecture are estimated to be 1.25, 2.15, and 1.90, respectively. So, the graphene on the SiN shows the highest normalized resistance sensitivity, while the graphene on SiO_2_/Si is the lowest one.

**Figure 5:**
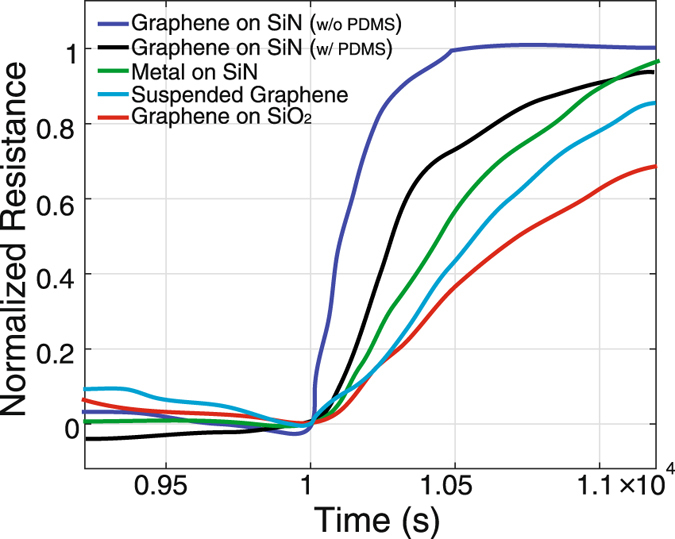
Transient response of the graphene temperature sensors with 5 °C temperature change is compared with thin film (40 nm Ni) RTD results in a temperature controlled test environment. The graph shows the substrate effect on response of SLG temperature sensor. The surface property effect on sensitivity combined with the thermal time constant effect on the dynamic behavior of the graphene temperature sensor is demonstrated.

Furthermore, the effect of the substrate on the device temperature transient response for five different configurations have been investigated as shown in Fig. 5. These responses can be explained in terms of thermal conductivity and thermal mass. All of the samples were covered with a PDMS gasket except the one sensor showing the fastest temperature response. The graphene on the SiN membrane without the PDMS gasket shows the fastest temperature response time. This is attributed to the low thermal mass of the SiN membrane, which equilibrates with the environment temperature. Since the thermal conductivity of graphene (5000 W/mK) is higher than that of SiN (4 W/mK)^16, 50^, the temperature response of the graphene follows the response of the SiN membrane. The response of the graphene on the SiN membrane is slower than that of one without the PDMS due to the PDMS thermal mass. The metal sensor made of a 100 nm-thick nickel film on the SiN membrane shows the third fastest response. Compared to graphene, the metal layer has a higher thermal mass, causing a slower response than the graphene layer. The suspended graphene sensor shows the second slowest temperature response. The high thermal conductivity of graphene is expected to lead to a fast response to environmental temperature changes. However, the graphene sensor is suspended across the Si substrate with a suspended length of 20 *μ*m equilibrates with the substrate, causing the slower response. This is because the substrate was mounted on an acrylic plate, which has a higher thermal mass. The slowest response is shown for the graphene sensor on the SiO_2_/Si substrate mounted on an acrylic plate due to having the highest thermal mass among the five configurations. The graphene temperature sensor on the SiN membrane has not only the fastest response, but also better mechanical stability compared to the suspended graphene sensor. Therefore, the optimal configuration of a graphene based temperature sensor would be the graphene sensor on a SiN membrane.

**Figure 6:**
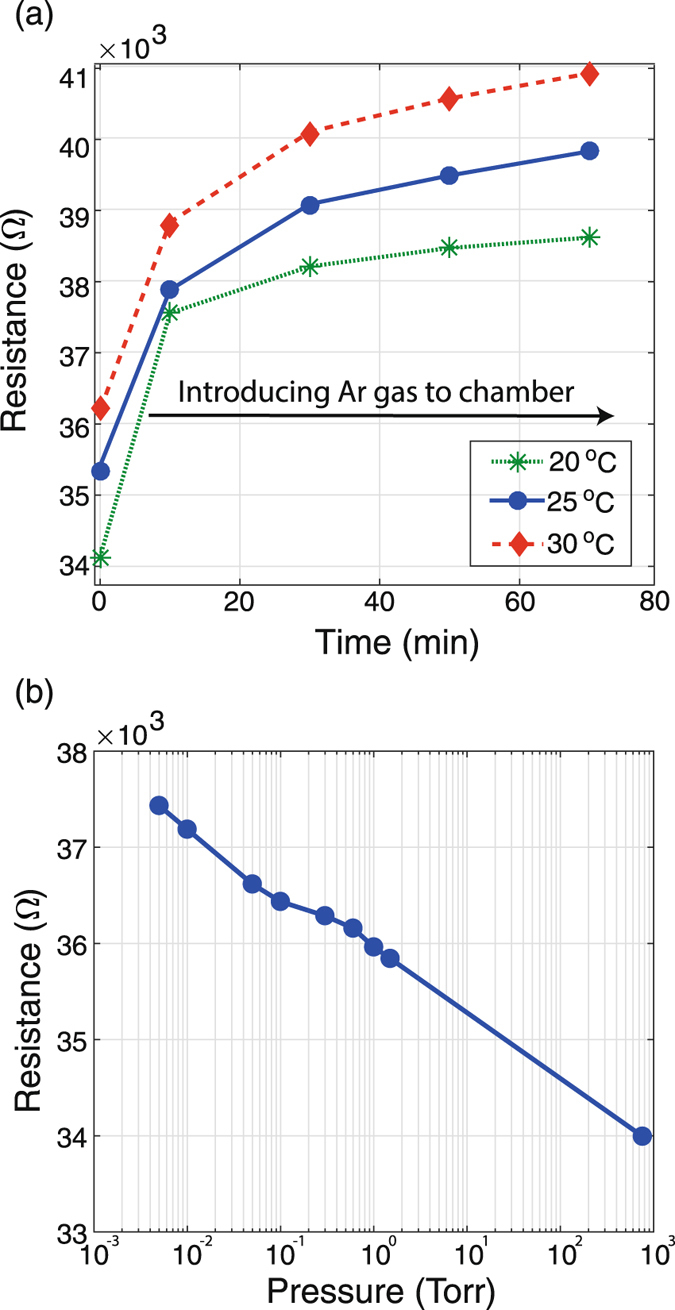
Graphene resistance affected by environments. (**a**) In an inert gas (Ar) environment. The resistance of the graphene increases as the air is replaced by Ar in the chamber and also as the temperature increases. (**b**) In vacuum pressure at room temperature. The lower pressures results in higher resistances.

The graphene temperature sensor can be affected by surrounding environments. For example, the humidity modulates the resistance of the graphene sensor^51^ as shown in Fig. 6. We measured the resistance of the graphene in an inert gas environment at 3 different temperatures (20, 25, and 30 °C) and vacuum at room temperature. To create an inert atmosphere, Ar gas, 20 *sccm*, was continuously flown through a chamber (400 *cm* ^3^), where the sensor is placed. Figure 6(a) shows that the resistance gradually increases and saturates after 70 minutes of Ar flow to the chamber. Until 30 minutes, the ambient gas is gradually replaced by the Ar gas. After 30 minutes, the chamber is saturated with Ar gas, and the resistance is saturated as well. The resistance increase is due to the lack of water molecules on the graphene sensor. Water molecules can shift impurity bands and modify their hybridization with graphene bands. This phenomena results in doping of the graphene layer^52^. Besides the doping effect, the resistance of the graphene sensor increases at the elevated temperatures as shown in Fig. 6(a). The resistance change in Ar environment is different from air environment, since the doping effect is reduced by replacing the air with Ar gas. As a result, the mobility of the graphene in Ar shows a different trend from the air case. The mobility as a function of temperature in Ar environment needs further investigation. To investigate the effect of water molecule concentrations on the graphene resistance change, the resistance is measured in a controlled vacuum as shown in Fig. 6(b). By decreasing the pressure the amount of water molecules on the graphene sensor decreases and results in higher resistances than that measured at ambient (due to the lack of water molecules and lower doping level).

## Conclusion

Micro-patterned SLG devices were fabricated and characterized as temperature sensors, showing the characteristics of temperature response between those of metals and thermistors. The quadratic dependence of resistance on the temperature for the fabricated graphene sensors was analyzed using the temperature-dependent carrier density and electron mobility model. The graphene sensor placed on the SiN membrane reveals not only the fastest response and the highest sensitivity due to the low thermal mass, but also better mechanical stability compared to the suspended graphene sensor. As a result, the suggested SLG on a SiN membrane can be an efficient temperature sensor for use in various fields requiring highly sensitive and fast operation.

## Experimental Section

### CVD Growth of SLG

The CVD reactor we used is a horizontal quartz tube, which is 2400 mm long and 152 mm in diameter. This quartz tube was placed in a 1500 mm long six-zone furnace. A 25 *μ*m-thick copper foil (from Alfa Aesar, 99.999%, 10 × 30 cm^2^) was loaded in the CVD reactor and pumped to the base vacuum pressure (<10^−4^ Torr). The copper foil was vertically placed on a substrate holder in the central isothermal growth zone of the reactor during the growth process. The temperature of the chamber was ramped up to 1060 °C with a mixture of Ar (2000 sccm) and H_2_ (30 sccm) at the processing pressure of 470 Torr. The copper foil was typically annealed for 2 hours. The furnace temperature was then decreased to the process temperature of 1020 °C with pure 30 sccm H_2_ gas only at 570 mTorr. When the temperature reached 1020 °C, graphene layers started to grow on the copper foil when a methane/hydrogen gas mixture (CH_4_ = 40 sccm and H_2_ = 100 sccm) and 2000 sccm Ar as a carrier gas were introduced to the reaction chamber at 600 mTorr for 30 min. The reaction chamber was then cooled down to room temperature with an average rate of 14 °C/min under the same Ar/H_2_ gas flow rates without methane.

### Transferring Graphene to a Substrate

**Figure 7:**
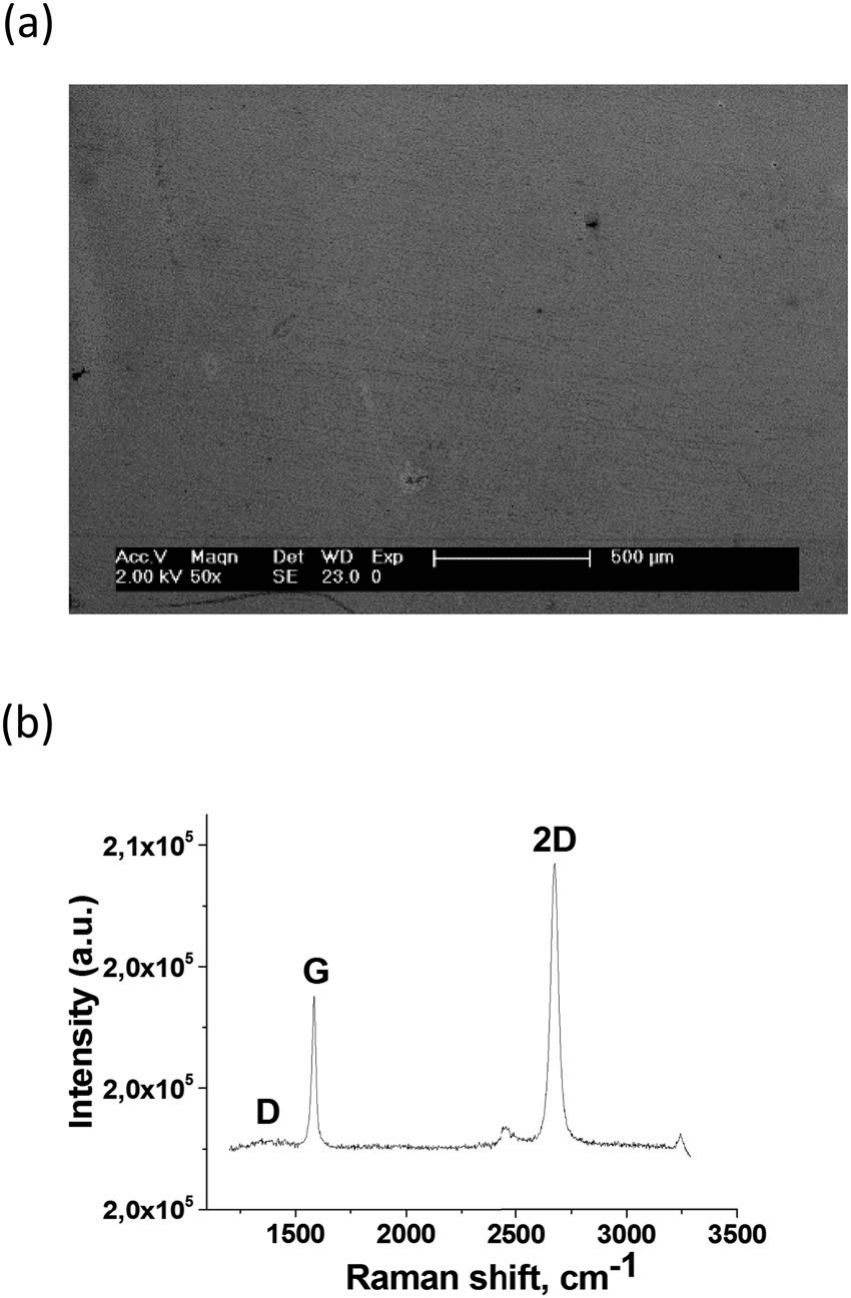
A single layer graphene transferred onto a SiO_2_/Si substrate. (**a**) The SEM image of a transferred graphene layer, (**b**) the Raman spectrum of a single layer of graphene with the peak intensity ratio of the 2D band to the G band greater than one, common for one layer of PMMA-transferred graphene^54^. The weak D band around 1350 cm^−1^ confirms the synthesized graphene is a high-quality layer.

A 2 *μ*m-thick PMMA (e-beam resist, 950 k C4, Microchem) was spin-coated on the as-synthesized graphene on copper foil at 3000 rpm for 100 seconds. The PMMA was baked in an oven at 120 °C for 10 minutes. Because the CVD process grew graphene layers on both sides of the copper foil, graphene layers the other side (no PMMA) of the copper foil were removed by oxygen plasma (60 W power for 10 minutes). The copper foil was then removed by floating the sample on a copper etchant (CE-100, commercially available from Transene) for 40~60 min. To rinse the graphene after copper etching, the PMMA/graphene was removed from the etchant and placed in two subsequent DI water baths for 20 min each. After the DI water rinsing, the PMMA/graphene layer was transferred onto a SiO_2_ (300 nm)/Si (100) wafer. Then, the PMMA was removed by acetone in an ultrasonic bath for 20 min, leaving the SLG on the SiO_2_/Si wafer. The single layer graphene on the SiO_2_/Si wafer was then rinsed in a 30% HCl solution at 60 °C for 30 min to remove residual Fe^+3^ ions. As a result, the high-quality graphene layer with a low defect density was prepared on the SiO_2_/Si substrate on a large scale (Fig. 7)^53^.

### Device Fabrication

The transferred graphene layers on SiO_2_/Si, a silicon nitride (SiN) membrane, and a silicon wafer with etched rectangular pits were patterned by a photo-lithography process. During the lithography process, the photoresist (PR) remaining on the graphene was removed by acetone after patterning the graphene. Since the surface and parts of the graphene were damaged during the removal of the PR, the PR on patterned graphene was not removed. Then, this remaining PR was used to support the suspended graphene over the pits (Fig. 1(c)). Without the PR support, the critical point drying process was needed to suspend the graphene.

### Raman Measurement

Graphene layers grown on Cu foil by the CVD method were transferred on the SiO_2_ layer and identified by a CRM 200 (WiTec, Germany) with a 100X lens (Olympus, N. A. 0.9) and a 532 nm laser (~1 mW) polarized by a GX-AN360 (Olympus) filter. Each spectrum was obtained by 10 acquisitions in 10 s of accumulation time. Three to six analyses were performed at various places on each sample.

### Electrical Measurement

Electrical contacts to external electrical measurement units were made using silver paint (TED Pella, Colloidal silver liquid, 16034) and 40 AWG copper wire (MSC Industrial supply co., 73225534) on the graphene contact pads in a wire bonding fashion. It should be noted that typical silver epoxy (TED Pella, 16014) may dissolve or make a poor electrical contact with the graphene based on the observations during the experiments. Despite this occasional malfunction, the 4-wire measurement eliminated any lead resistance (graphene traces and conductive epoxy) from contributing to the temperature sensor resistance.
